# Miniaturized Sample Preparation and Rapid Detection of Arsenite in Contaminated Soil Using a Smartphone

**DOI:** 10.3390/s18030777

**Published:** 2018-03-04

**Authors:** Mohd Farhan Siddiqui, Soocheol Kim, Hyoil Jeon, Taeho Kim, Chulmin Joo, Seungkyung Park

**Affiliations:** 1 School of Mechanical Engineering, Korea University of Technology and Education, Cheonan 31253, Korea; mohdfarhan@koreatech.ac.kr (M.F.S.); wjsgydlf@koreatech.ac.kr (H.J.); taeho4111@koreatech.ac.kr (T.K.); 2 School of Mechanical Engineering, Yonsei University, Seoul 03722, Korea; improvely@naver.com

**Keywords:** biosensor, soil arsenic monitoring, miniaturized, colorimetric detection, smartphone

## Abstract

Conventional methods for analyzing heavy metal contamination in soil and water generally require laboratory equipped instruments, complex procedures, skilled personnel and a significant amount of time. With the advancement in computing and multitasking performances, smartphone-based sensors potentially allow the transition of the laboratory-based analytical processes to field applicable, simple methods. In the present work, we demonstrate the novel miniaturized setup for simultaneous sample preparation and smartphone-based optical sensing of arsenic As(III) in the contaminated soil. Colorimetric detection protocol utilizing aptamers, gold nanoparticles and NaCl have been optimized and tested on the PDMS-chip to obtain the high sensitivity with the limit of detection of 0.71 ppm (in the sample) and a correlation coefficient of 0.98. The performance of the device is further demonstrated through the comparative analysis of arsenic-spiked soil samples with standard laboratory method, and a good agreement with a correlation coefficient of 0.9917 and the average difference of 0.37 ppm, are experimentally achieved. With the android application on the device to run the experiment, the whole process from sample preparation to detection is completed within 3 hours without the necessity of skilled personnel. The approximate cost of setup is estimated around 1 USD, weight 55 g. Therefore, the presented method offers the simple, rapid, portable and cost-effective means for onsite sensing of arsenic in soil. Combined with the geometric information inside the smartphones, the system will allow the monitoring of the contamination status of soils in a nation-wide manner.

## 1. Introduction

Arsenic is the 20th most common naturally occurring element in the Earth’s crust, found both in the soil and water. Due to its toxic nature, it poses a great threat to the living organisms [1,2,3]. Even in traces, it can affect cardiovascular, respiratory, neurological systems in general and acute risk of lung, bladder and skin cancers in particular [4,5]. Various cross-sectional studies reported As(III) contamination (in drinking water) related casualties across the globe especially, from Poland, Canada, Taiwan, India, Vietnam and Bangladesh [1,6,7,8,9,10]. However, these reports are limited to the drinking water only, and less is known about the effects of arsenite [As(III)] contamination in the soil. Moreover, previous literature suggested a much higher concentration of As(III) in the soil as compared to the water [11]. Arsenic concentration in the soil fluctuates widely, from 1 to 40 mg/kg (ppm, parts per million), with an average level of 3–4 mg/kg. World Health Organization (WHO) and U.S. Environmental Protection Agency (EPA) proposed stringent guidelines allowing the maximum permissible limit of arsenic in the soil below ~5 mg/kg [12]. These findings suggested that soil contamination could be a major threat to human health due to biomagnification, bioaccumulation, and biotransference [13,14,15]. Thus, on-site detection of As(III) level in the soil is crucial to prevent any toxicological repercussions.

Arsenite (III) and arsenate (V) are the two key inorganic forms which are harmful to the organism, arsenite (III) being more toxic [16,17]. Under reducing conditions, arsenite forms various toxic compounds through biochemical modification and causes severe toxicity by inhibiting many crucial enzymes and proteins involving in DNA synthesis and repair system [18,19]. Arsenic toxicity is not only limited to animals, but it is phytotoxic too [20]. The primary cause of arsenic contamination in the soil is mining, industrial waste deposition, and application of fertilizers and chemicals, thereby inducing secondary contamination in the air or water by wind blowing, leaching, and weathering [21,22,23,24,25].

There are a number of conventional analytical methods for accurate detection of arsenic in the environment such as inductively coupled plasma mass spectrometry (ICP-MS), hydride generation atomic absorption spectroscopy (HG-AAS), atomic fluorescence spectroscopy (AFS), and neutron activation analysis (NAA), with an extremely high sensitivity up to 0.5 µg/L (0.5 ppb) [26,27,28,29]. These methods have high accuracy and sensitivity, but perhaps the biggest disadvantage of these methods is the requirement of complicated sample preparation steps, expensive and bulky equipment, skilled personnel and safety standards in a central laboratory. Furthermore, all aforementioned methods are time-consuming, require high maintenance, and offer offsite detection only [30]. Meanwhile, there are some convenient arsenic detection field kits based on Gutzheit and Hach methods, which have shown the feasibility of water-based analysis, but the risk of toxic arsine gas release remains a concern [31,32]. To further alleviate the problems, several miniaturized optical, electrochemical and biological sensors have been proposed [33,34,35,36,37,38]. These sensors have shown the alternative ways of detection for designated target ions with reasonable effectiveness and sensitivity but imposed several limitations such as fluorescence dependence, sophisticated electrode preparation, and tedious bioassay with low stability [17,39,40,41,42,43].

The high affinity, stability, and specificity of aptamer act as a powerful tool for microfluidic detection of biomolecules. Recently, researchers have employed aptamer-based techniques on arsenic detection too [44]. Especially, several researchers utilize a combination of aptamers and gold nanoparticles (AuNPs) for relatively simple, reproducible and sensitive colorimetric detection of arsenic As(III) in water [42,45,46,47,48]. While achieving the simple and sensitive detection in water, the use of toxic reagents like silver, CTAB and PDDA, dependence on sophisticated external optics (UV spectrophotometer, microplate reader and circular dichroism), time-consuming functionalization and characterization steps of gold nanoparticles still require laboratory-based processing [45,49,50]. Moreover, there is a paucity of the on-site detection of arsenic in soil, and the functionality of available As(III) detection kit for water samples is yet to be validated for the soil. Unlike water, soil samples demand complicated pre-processing steps for serial extraction and purification, followed by optimization of the detection assay in sample matrices [51]. Thus, despite its simplicity and sensitivity, the aptamer-based methods are restricted to laboratory-oriented analysis.

Apart from the drawbacks of the above-mentioned methods, portability is another critical factor for on-site detection. The portability of analytical system should be emphasized on resource-limited settings where power supply, skilled operators, and laboratory setup are lacking. Currently, the omnipresent smartphones are the most suitable means for providing the portability. Combined with data processing, analysis, and geometric data communication abilities, smartphones can be an ideal platform for the contamination monitoring of the soils in large areas. Certain reports involving colorimetric detection of analytes with data processing and sharing facility are available [52,53,54,55]. Nevertheless, there is absentia of mobile platforms for arsenic detection in the soil and the automation of analytical procedures.

**Figure 1:**
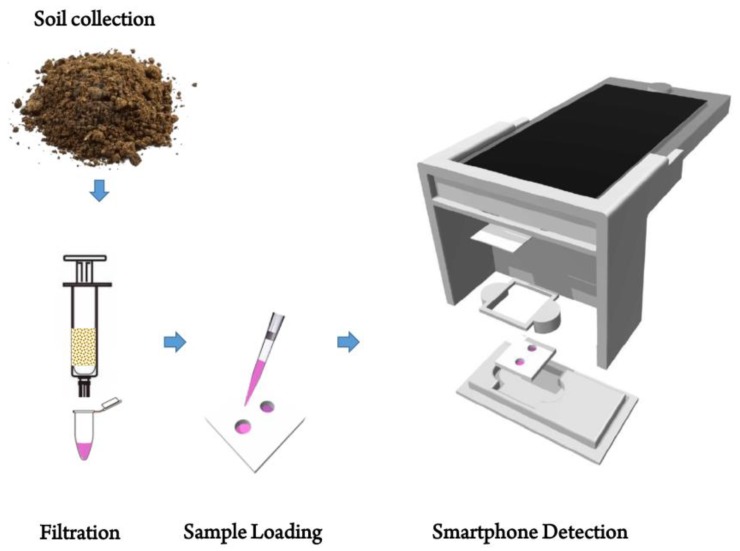
Schematic illustration of soil processing and smartphone-based arsenic detection procedure.

This paper, for the first time to our knowledge, presents a smartphone-based portable system for rapid, simple, and cost-effective sample preparation and detection of arsenic As(III) in the soil. An aptamer and AuNPs based colorimetric detection assay has been developed for the stability and the simplicity of optical detections through a smartphone. The detection assay has been designed in a reduced volume configuration (50 µL) for minimizing complexity and cost of analysis and optimized for the colorimetric detection under the smartphone optics. Figure 1 shows the overall analytical process.

## 2. Experimental Section

### 2.1. Chemicals and Reagents

Arsenic As(III) binding aptamer (ARS-3) (5′GGTAATACGACTCACTATAGGGAGATACCAG CTTATTCAATTTAAGAACAACCAACGTCGCTCCGGGTACTTCTTCATCAGATAGTAAGCAATCT-3′) is synthesized and purified by polyacrylamide gel electrophoresis by Bioneer (Daejeon, Korea). HEPES buffer (50 mM, pH 7.2) is purchased from Sigma-Aldrich (St. Louis, MO, USA), to dissolve aptamer. The monodisperse citrate-capped gold nanoparticles with the diameter of 15 nm are obtained from Nanopartz (Loveland, CO, USA). Sodium chloride (NaCl) and Sodium hydroxide (NaOH), used as a salt and bases respectively, are ordered from Merck (Darmstadt, Germany). Standard solutions of arsenic and other heavy metal ions (Pb, Hg, Ni, Cu, and Fe) (1 mg/mL, 1000 ppm) are purchased from Kanto Chemicals (Seoul, Korea). Hydrochloric acid (HCl) is acquired from Samchun Chemicals (Seoul, Korea).

### 2.2. Extraction Procedure for Arsenic

The standard protocol for arsenic extraction from soil is based on strong acids such as aqua regia, but mild acids based protocol is also known to be effective for mobile arsenic As (III), which is more toxic to the humans than the bound form [56]. Because of comparable efficiency, while providing the ease of handling in fields, the soil extraction in this study has been performed with 1 M HCl. 3 mg of soil samples are added to 20 mL of 1M HCl and mixed for an hour, and then filtered through a filter-paper (pore size of 12 µm) in a syringe. The extracted solution is then loaded for further analytical steps.

### 2.3. Fabrication of PDMS Chip

The PDMS offers inertness and transparency which is a pre-requisite for any chemical reaction based image analysis [57]. The PDMS mixture (Sylgard 184, Dow Corning Corporation, Midland, MI, USA) in 10:1 ratio of monomer and curing agent was prepared. The mixture was mixed properly in the disposable plastic dish for 15 min. Thereafter, we poured the mixture into the petri plate and degassed it for 2 h. After degassing in a vacuum chamber, the mixture was left overnight at room temperature for polymerization. Subsequently, the polymerized PDMS was peeled off and cut into pieces of size 22 mm × 22 mm. These pieces were punched to form wells of various sizes 2.5, 4, 6, and 8 mm in diameter and 8, 6, 4, and 2 mm in thickness, respectively. Finally, the punched PDMS was fixed on a glass coverslip, and a white styrofoam tape of size 22 mm × 22 mm was attached at the back of the coverslip. The overall dimensions of sandwich assembly were optimized several times to achieve optimum resolution and avoid shadows in corners; deeper/smaller wells cast shadows in the corner and give darker images which cause difficulties in image processing (Figure S1).

### 2.4. Arsenic Detection Standard Protocol

Standard protocol is designed according to the miniaturized sample volume on the optical platform. Optimization of salt and aptamer concentration for a fixed amount of AuNPs is important to finalize the protocol for a miniaturized PDMS chip platform. The total working volume of the system is fixed at 50 µL consisting of AuNPs, ARS-3 aptamer, arsenic sample and NaCl. Firstly, the aptamer is dissolved in HEPES buffer (50 mM, pH 7.2) at 95 °C for 5 min. Then, the concentrations of NaCl and aptamer for a fixed amount of AuNPs (35 µL) are optimized. After setting the concentration of NaCl and aptamer, the whole standard protocol is finalized for 50 µL of working volume. The designed standard protocol is then validated with the arsenic samples in neutralized solution (1 M HCl + 1 M NaOH, pH 7.0). The sample is prepared by mixing 5 µL of aptamer (20 nM) with 35 µL of AuNPs (2 nM, 15 nm) and dispensing into the PDMS-chip platform. Subsequently, the chip is covered with a cover slip and incubated for 10 min at the room temperature. After the incubation, 5 µL of arsenic As(III) spiked solution at an appropriate concentration is introduced into the mixture and the PDMS-chip is covered and incubated for 7~10 min. Finally, 3 µL of NaCl (0.4 M) is added to the above mixture, and then the chip is transferred to the cartridge for the smartphone-based colorimetric analysis. Thereafter, the optimized assay was performed with the standard arsenic solution at various concentrations (0.0–40 ppm) and repeated five times. The data obtained was utilized for the preparation of standard curve and obtaining the limit of detection. To confirm the standard protocol, spiked soil samples were prepared. Field soils that contain other heavy metals ions and organic components except for the arsenic ions were collected, dried and sieved. These soils were then spiked with three concentrations of arsenic (5, 15, and 20 ppm) (following the guidelines of analytical detection [58]). All the spiked soils are tested three times. Images were obtained, analyzed and results were compared with ICP-MS data.

#### 2.4.1. Salt Concentration Optimization

**Figure 2:**
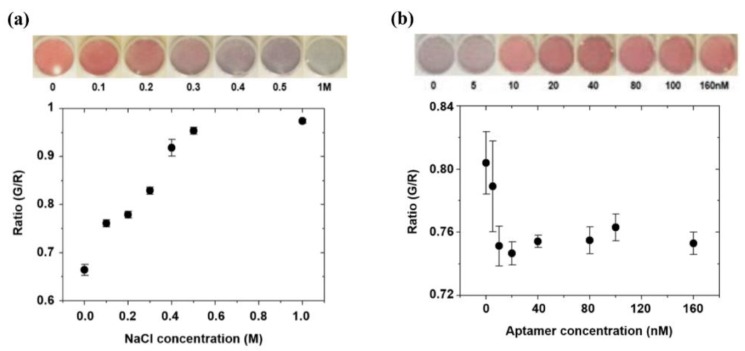
(**a**) Colorimetric intensity variation (ratio of green to red) at different NaCl concentrations required for optimum aggregation of 2 nM of AuNps; (**b**) Colorimetric intensity variation (ratio of green to red) at different aptamer concentrations.

Optimization of salt (NaCl) is crucial to generate the standard protocol for colorimetric detection because the degree of aggregation of AuNPs depends on salt concentration. If the salt concentration is too high in the AuNPs solution, the aggregation may be too intense or vice-versa, which will result in instant decolorization. The optimum NaCl concentration for a fixed amount of AuNPs aggregation has been studied as shown in Figure 2a. The ratio of green to red color intensity (G/R) is utilized to quantify the degree of aggregation at known concentrations of NaCl. The color intensities are evaluated from triple set of experiments for each concentration. As per the Figure 2a, G/R value is increasing with increased NaCl concentration, and getting saturated, indicating that the concentration below 1 M is sufficient for complete aggregation of nanoparticles. Based on the developed color intensity, 0.4 M NaCl has been selected as an optimum concentration for complete aggregation of 35 µL of 2 nM of AuNPs. It should be noted that higher concentration than the selected optimum can be applied, but the aggregation of AuNPs and decolorization of the assay may be accelerated. The photographs in Figure 2a showed the circular test zones on the PDMS-chips at different NaCl concentrations (0~1 M).

#### 2.4.2. Aptamer Concentration Optimization

In general, increased aptamer concentration prevents the aggregation of a fixed amount of AuNPs in the presence of NaCl, keeping the AuNPs in a dispersed state. For the optimal competitive reaction, we have adjusted the ARS-3 aptamer concentration for 35 µL of 2 nM AuNPs, in the presence of the previously optimized NaCl concentration. The inset photographs show the color variation of the reaction mixture at different aptamer concentrations (0~160 nM). It can be observed that AuNPs are well dispersed from the aptamer concentration of 20 nM. In Figure 2b, G/R values with respect to the aptamer concentration are shown. Each data point is measured from triple set of experiments. The G/R value decreases as the aptamer concentration is increased (0~160 nM), but the intensity is stable above the concentration of 20 nM. Thus, the minimum aptamer concentration is selected as 20 nM, which is sufficient to prevent aggregation in the presence of NaCl.

### 2.5. Specificity Test for Arsenic As(III) Determination

The specificity of the developed assay for target ion, As(III), over other metal ions is investigated under the same experimental condition. In this test, standard solutions of other heavy metal ions which are expected in contaminated soil like Pb^2+^, Hg^2+^, Cu^2+^, Ni^2+^, and Fe^3+^ are prepared. Then, 20 ppm of each standard solution of heavy metal ions are spiked into the 1 M HCl + 1 M NaOH solution (pH 7.0). Every sample solution is adjusted to have the same amount of aptamer, AuNPs, and NaCl. The prepared mixture is dispensed into the PDMS chip. Thereafter, the color intensity is measured, and the concentration is predicted based on the calibration curve. Each test is repeated three times.

### 2.6. Image Acquisition & Design of Smartphone-Based Optical Device

**Figure 3:**
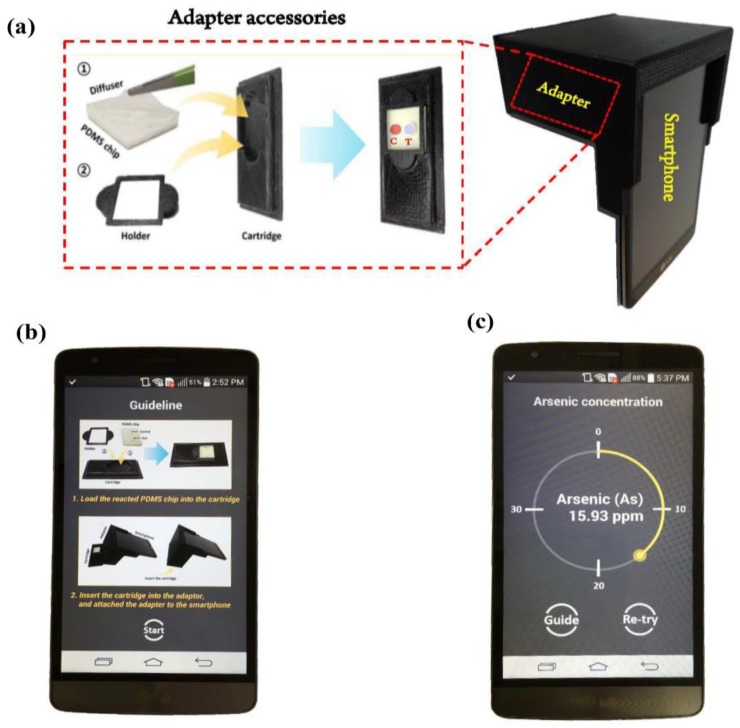
(**a**) Optical device with its accessories; (**b**) Screenshots of operating on an Android smartphone: Guideline of preparation for measurement of arsenic As^3+^ concentration; (**c**) The result of estimated arsenic As^3+^ concentration processed by Android OS using colorimetric analysis.

Colorimetric analysis and subsequent quantification of As(III) concentrations are performed via a smartphone-linked device. The basic operation involved illumination of the sample through a camera flash and collecting their resulting intensity in the form of a captured image. The processing of captured images with different intensity of color channel correlated the concentration of target analyte. In the present demonstration, as shown in Figure 3a, we employed a LG-F470L phone (LG Electronics, Inc., Seoul, Korea) as an optical reader. The phone is equipped with Android operating system (OS) platform (Android 4.0.3, IceCreamSandwich), a LED flash and an 8-mega-pixel CMOS image sensor, whereas the optical hardware unit consists of adapter, holder, cartridge, diffuser, and chip. The diffuser made up of a layer of 3M Scotch tape (810R-12, Elyria, OH, USA) is placed on top of the PDMS chip to ensure uniform LED illumination across the detection chip while alleviating the specular reflection from the liquid-air interface of the chip. All the components of the optical unit are designed with Solidworks 2010 (Solidworks Corporation, Concord, MA, USA) and fabricated with a 3D printer (Measurement Korea Corp., Cheonan, Korea, Wiiboox 3D printer). Black poly lactic acid (PLA) is used as the material. The cartridge (75 mm × 35 mm) is made to securely position the PDMS-based detection chip (22 mm × 22 mm) so that it can be located in the field-of-view of smartphone image sensor. The cartridge has a square socket of size 25 mm × 25 mm with the holder for placing and fixing PDMS chip inside the cartridge and can be inserted in the designated plate in the smartphone adapter. The function of smartphone adapter is to block the ambient light and hold the cartridge at a fixed distance (50 mm). The dimensions of the adapter are 75 mm × 35 mm × 65 mm (Figure 3a). The whole hardware unit weight is estimated to be ~55 g with total volume of PDMS chip wells of 50 µL. The PDMS chip has two circular zone control “C” (without arsenic in the sample) and test “T” (having different concentration of arsenic in the sample). The PDMS engraved wells have a diameter of 6mm and the depth of 4 mm, as indicated in Figure S1. Once this detection chip is loaded, the custom-built smartphone application acquired the image (8-bit) and cropped region of interest (ROI) with a size of 1.5 mm × 1.5 mm at the center of the measurement region. The RGB pixel values in the ROI are spatially averaged, and the ratio of the green to red values (G/R) is evaluated. We assessed various other color space models such as HSV and CMYK and found that the G/R ratio exhibited the highest correlation with the arsenic As(III) concentrations in the samples.

## 3. Results and Discussion

### 3.1. Android-Based Smartphone Application

Android application software (App) is developed for quantitative measurement of arsenic As(III) concentration with a smartphone (Figure 3c). Once initiating the App, users are provided with instructions on the installment of detection cartridge in the adapter (Figure 2b). After the detection cartridge is loaded and the adapter is attached to the smartphone, the users press the “Start” button to begin the measurement. The App then acquires the image, decomposes the image in red, green and blue colors and computes the ratio of the green to the red color (G/R). The evaluated value is compared against the calibration data (Section 3.4) to obtain the arsenic As(III) concentration. The overall time for image processing and computation is measured to be ~2.5 s. The acquired data can be saved as both an ASCII and an image (JPEG) file. Figure 3c shows the snapshot of the App display with the estimated arsenic As(III) concentration. Detection range is set to be 1 to 40 ppm in our application. If arsenic As(III) concentration is measured beyond the detection range, an error message is displayed on the screen.

### 3.2. Colorimetric Detection Mechanism of Arsenic As(III) and Standard Calibration Test for Arsenic As(III) Detection

**Figure 4:**
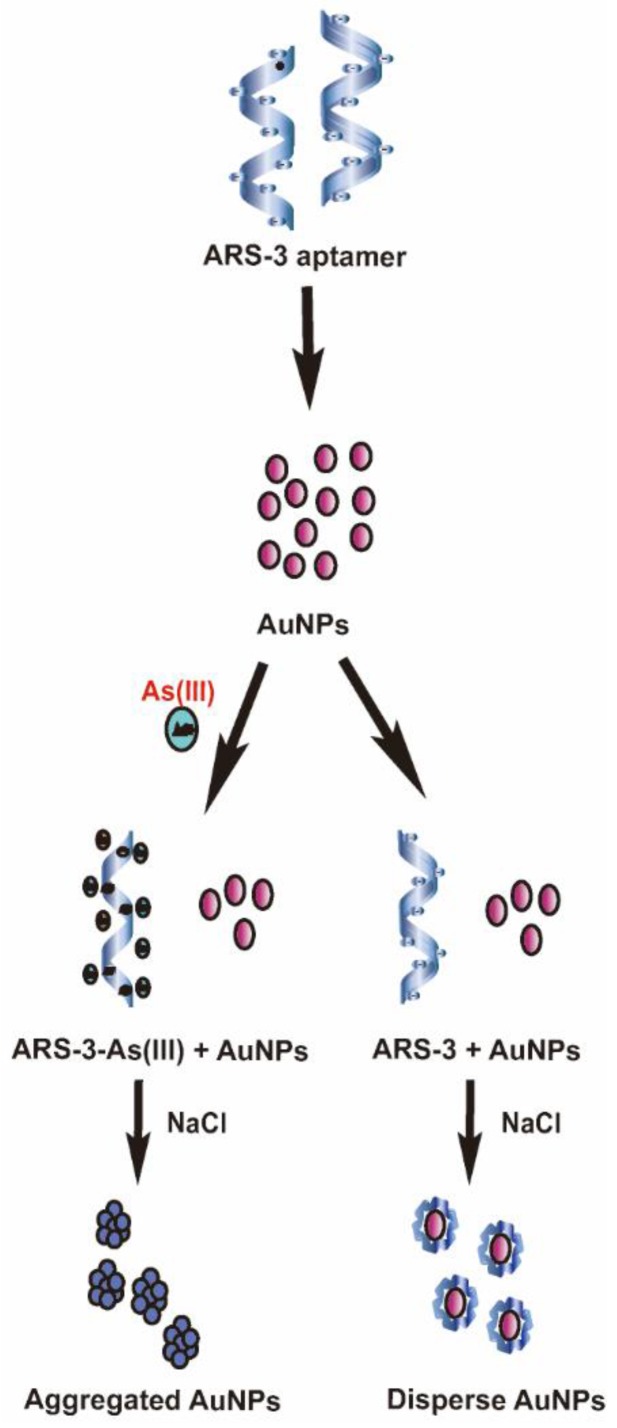
The principle of arsenic As^3+^ detection with AuNPs.

AuNPs have been widely used as a sensing tool for biological application because of broad-spectrum color exhibiting property [56]. Based on the aggregation and colorimetric property of AuNPs, several methods have been designed for sensitive sensing including arsenic detection in water [57]. The basic approach of AuNPs-aptamer method explores the specific binding affinity of ARS-3 aptamers molecules to the arsenic As(III) ions. During NaCl treatment, in the absence of arsenic As(III), ARS-3 aptamer stabilizes the AuNPs covering its surface through electrostatic interaction whereas the interaction is distorted in the presence of the arsenic molecule, resulting in the formation of ARS-3 and As(III) complex. As a result, the AuNPs destabilized and aggregated at high concentrations of NaCl, induced the color change from red to grey (Figure 4). Compared to arsenic detection in water which is neutral, soil samples need to be treated with acids to extract arsenic ions. The change in pH and ionic strength due to acid and salt influences the conformation of aptamer [58], affecting the overall sensitivity of the device. Thus, the previously developed protocol for water cannot be applied directly to the soil and need to be modified to maintain the compatibility with soil solution. We have used mild acid (1 M HCl) to extract the As(III) from the contaminated soil which decreased the pH of the sample causing the protonation of the aptamer. Therefore, in order to maintain the pH, we have neutralized the sample with base (NaOH) before proceeding the experiment.

**Figure 5:**
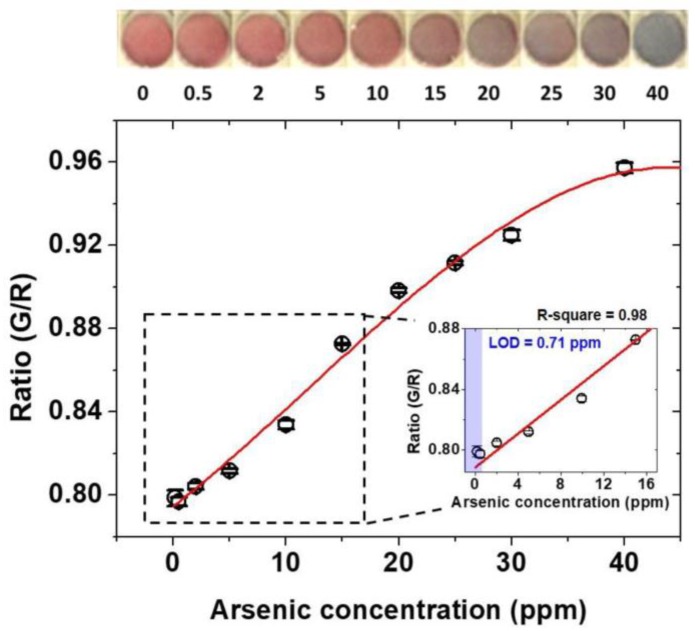
Standard calibration curve represented the G/R ratios measured at different arsenic As(III) concentrations (black empty circles), along with the fitted standard curve (red line). Each black empty circle related to the standard deviation (error bars) of five repeated experiments.

The limit of detection of any image-based system is dependent on the limit of resolution of the captured images. Hence, the fabrication of a PDMS chip was crucial step to achieve the maximum resolution in the images. Small diameter and thickness (2.5 mm × 8 mm, 4 mm × 6 mm) lead to shadow effect in the corners and reflections (Figure S1), while bigger diameter and thickness (8 mm × 4 mm) results in low color intensity (Figure S1). The optimum dimensions should not have shadows and reflections, the diameter of 6 mm and thickness of 4 mm are found to be most appropriate for the image-based analysis. Our results, with the modified protocol and optimized PDMS chips, indicate a smooth transition from red to the gray color on increasing the arsenic concentration (Figure 5). Thus, the optimized protocol is feasible for neutralized soil extracts, same is supported by data obtained from the spiked soil samples and hence could be a promising tool for the detection of the real soil samples.

To assess the utility of the device for quantitative analysis of arsenic As(III) in the soil, the standard solutions with known concentrations of arsenic As(III) (0 ~ 40 ppm) are spiked in neutralized extractants having aptamer, AuNPs, and NaCl. The use of spiked soil samples is a general practice to assess the utility of the device on real soil [58]. The extract of spiked soil samples includes the other ion as well as the organic contaminants. The high coefficient of correlation is an indicative of a minimum interference of the organic and inorganic contaminants. All samples are assessed visually using smartphone device within ~30 min and tested with control samples to minimize the uncertainty in the results. Figure 5, shows the quantification of G/R intensity experimentally obtained with increased arsenic As(III) concentration along with the fitted standard curve. The standard curve was fitted with MATLAB software program (R2017a, MathWorks, Inc., Natick, MA, USA). The intensity increases with the increase in the arsenic concentration and shows the saturation point beyond the 40 ppm arsenic. Inset photographs show the color change of the assay for each case. As the arsenic concentration is getting higher, overall color is shifted from red to grey, indicating the increased aggregation of AuNPs in the presence of arsenic As(III). The color remained red near 0.5 ppm because the amount of arsenic As(III) is not sufficient to break the electrostatic interaction between aptamer and AuNPs. The limit of detection (LOD) is calculated by taking the ratio of standard deviation of control samples ($\sigma_{c}$) (samples without arsenic), over the sensitivity, or the slope of the standard curve at a low arsenic concentration ($S_{std}$). The inset graph shows a linear response in the range of 0~15 ppm with the coefficient of determination of 0.99177 (R-square). The coefficient of determination was calculated with the functions in Curve Fitting Tool box in MATLAB. The resulting LOD of As(III) in the sample is measured to be 0.71 ppm. Reproducibility of the experiment is confirmed by the repetition in five times, which can be depicted from corresponding error bars with each black empty circle in the graph. The overall results demonstrate the accuracy of the protocol to detect the arsenic As(III) with LOD less than the USEPA recommended a limit of arsenic As(III) in the soil.

### 3.3. Detection of Arsenic As(III) in Spiked Soil Sample

**Figure 6:**
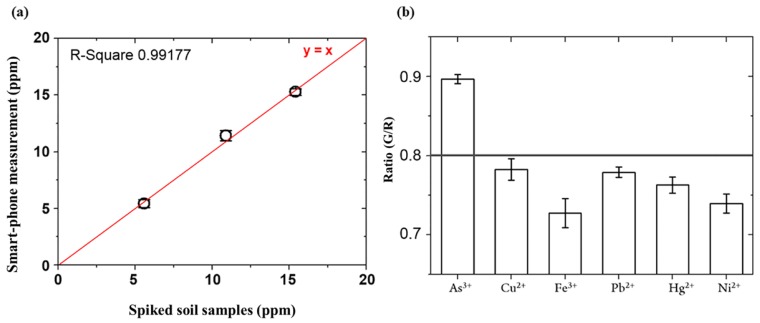
(**a**) Comparison of smartphone-based measurement with the standard method (ICP-MS) for arsenic As(III) spiked soil samples at different concentrations; (**b**) Specificity test for arsenic As(III) against other heavy metal ions at the same concentration (20 ppm). Each column related to the standard deviation (error bars) of three repeated experiments.

The analytical results are compared with the presented smartphone-based detection system. Figure 6a represents the comparative analysis of smartphone-based measurements for the spiked soil samples. The measurement results showed a good agreement with the correlation coefficient of 0.9917 and the average difference of 0.37 ppm. High coefficient of correlation with the standard equipment is an indicative of the accuracy of the current device, negligible interference with organic and inorganic components, and thereby, its real-world applications.

### 3.4. Specificity Test for Arsenic As(III) in the Presence of other Heavy Metal Ions

Pb^2+^, Hg^2+^, Cu^2+^, Ni^2+^, and Fe^3+^ are readily present contaminant in the soil. It was mandatory to establish the specificity of ARS-3 aptamer towards the As(III) in the presence of common heavy metal contaminants. Figure 6b shows G/R intensity values measured by the smartphone system for various heavy metal ions. It can be observed that only As(III) shows higher intensity than the detectable value, which corresponds to the LOD. The G/R intensity values of other metal ions are below the LOD. The test results clearly demonstrate high specificity for arsenic As(III) in the presence of other heavy metal ions below 20 ppm level, and thus interference can be avoided in the range of detection.

## 4. Conclusions

In summary, we have developed a portable smartphone-based optical device that utilizes AuNPs and aptamer as a signal probe for monitoring of arsenic As(III) in the soil. The detection protocol is optimized for the miniaturized setup and smartphone optics. The system components include optical adapter, PDMS chip, and Android application are designed to provide simple analytical process. We have successfully validated the performance of the device through the test of arsenic As(III) detection at a different concentration, in standard and spiked soil samples. Subsequently, a comparative analysis with the standard method (ICP-MS) was performed. Conclusively, we are able to construct a reproducible, reliable and portable device with a LOD of 0.71 ppm, which is much less than the permissible range of arsenic contamination in the soil (5 ppm). In comparison with other traditional techniques, the developed device has the potential for rapid sample preparation and detection in less than 3 h, minimal use of reagents with the low working volume of 50 µL, and reduced cost of analysis around 1 USD including manufacturing cost of a chip. In addition, the whole process is managed with convenient light weight (~55 g) smartphone-based operating system that can be easily handled on-site by nonprofessionals. However, the applicability of device is yet to be tested with the real samples. Multiple-sample detection and automation are other areas to cover in the future. In the long term, our proposed method will allow the continuous monitoring of arsenic contamination status even in resource limited area by providing spatial mapping and its database. Moreover, the proposed portable system can also be used for water analysis by optimizing the protocol for neutral samples on a miniaturized condition. In future trials, the performance of the device will be further validated and optimized through field tests at multiple sites to provide robust sensitivity and selectivity for various types of soils.

## Supplementary Materials

The following are available online at http://www.mdpi.com/1424-8220/18/3/777/s1, Figure S1: (**I**) (a) Dimensions of PDMS chip, (b) Fabricated PDMS chip. (**II**) Different chip designs with control (C) and test (T) wells, showing diameter, length, breadth and thickness: (a) 2.5, 22, 22, and 8 mm; (b) 4, 22, 22, and 6 mm; (c) 6, 22, 22, and 4 mm; (d) 8, 22, 22, and 4 mm. (**III**) Images of chips tested on the device, representing: (1) Reflection with shadow effect; (2) Shadow effect at the corners; (3) High intensity with no reflection and shadow effect; (4) Reflection with poor intensity.
