# Supplementary material for: Miniaturized Sample Preparation and Rapid Detection of Arsenite in Contaminated Soil Using a Smartphone
Source: Sensors (Basel). 2018 Mar 4;18(3):777. doi: 10.3390/s18030777 (PMC5877113; doi:10.3390/s18030777)
Supplement: Supplementary file 1 [file sensors-18-00777-s001.pdf]

Supplementary Materials: Miniaturized Sample Preparation and Rapid Detection of Arsenite in Contaminated Soil Using a Smartphone

Mohd Farhan Siddiqui[1], Soocheol Kim[2], Hyoil Jeon[1], Taeho Kim[1], Chulmin Joo[2*], Seungkyung Park[1*]

[1]School of Mechanical Engineering, Korea University of Technology and Education, Cheonan, 31253, South Korea

[2]School of Mechanical Engineering, Yonsei University, Seoul, 03722, South Korea

[*]Corresponding authors: cjoo@yonsei.ac.kr, Tel. +82-2-2123-5822, Fax. +82-2-312-2159; spark@koreatech.ac.kr, Tel. +82-41-560-1149, Fax. +82-41-560-1253

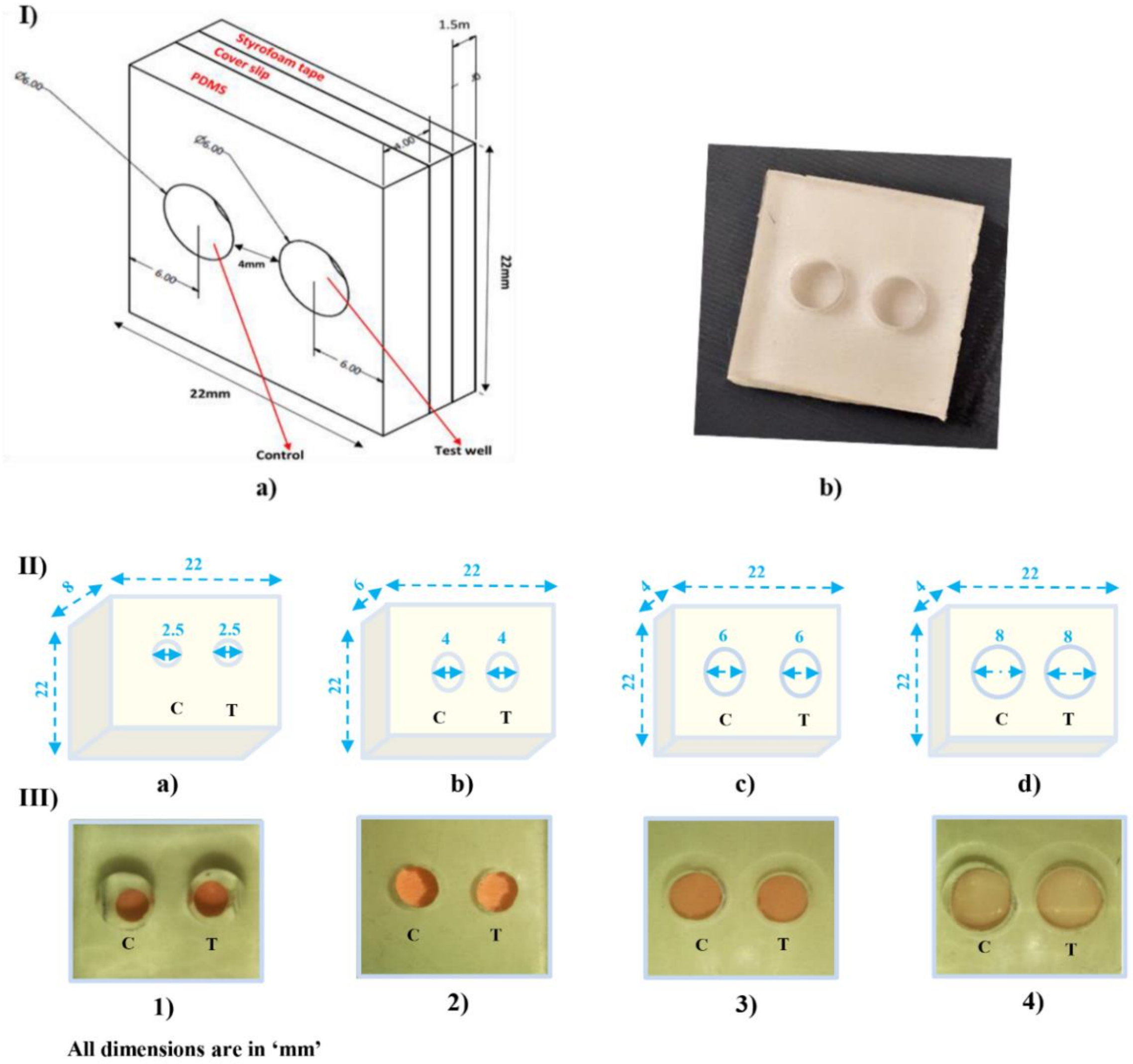

Figure S1: I) a) Dimensions of PDMS chip b) Fabricated PDMS chip. **II)** Different chip designs with control(C) and test (T) wells, showing diameter, length, breadth and thickness: a) 2.5,22,22, and 8 mm. b) 4,22,22, and 6mm c) 6,22,22, and 4mm d) 8,22,22, and 4mm. **III)** Images of chips tested on the device, representing: 1) Reflection with shadow effect 2) Shadow effect at the corners 3) High intensity with no reflection and shadow effect 4) Reflection with poor intensity
